# Volitional Yoga Breathing for Smokers: Development, Validation, and Feasibility of a 30-Minute Breathing-Only Intervention for Smoking Cessation and Pulmonary Rehabilitation

**DOI:** 10.7759/cureus.101716

**Published:** 2026-01-16

**Authors:** Sunil Kumar, Sarashti Saini, Gautam Mishra, Abhijit Baishya, Shivam Sharma, Darshana Hazarika, Sanjib Patra

**Affiliations:** 1 Department of Yoga, Central University of Rajasthan, Ajmer, IND; 2 School of Science and Humanities, Swami Vivekananda Yoga Anusandhana Samsthana, Bengaluru, IND

**Keywords:** craving, lung function, nicotine dependence, pranayama, pulmonary health, smokers, smoking cessation, tobacco use, yoga breathing

## Abstract

Background

Cigarette smoking is a major preventable cause of morbidity and premature mortality. It is associated with respiratory symptom burden, impaired pulmonary function, and high relapse rates during quit attempts. Relapse is often driven by nicotine craving, stress, and emotional dysregulation. Volitional yoga breathing (VYB) has shown potential benefits for reducing craving, improving emotional regulation, and supporting pulmonary health. However, no standardized breathing-only intervention has been developed specifically for smokers. The purpose of this study was to develop and validate a structured 30-minute VYB module and assess its feasibility and acceptability among chronic smokers.

Methods

The study employed a three-phase design. Phase 1 involved the development of a structured VYB module based on classical and contemporary yoga texts, supported by relevant scientific literature. Phase 2 comprised expert validation by 32 qualified yoga professionals using a 3-point Likert scale, with content validity quantified using Lawshe’s content validity ratio (CVR). Phase 3 evaluated feasibility in 20 high nicotine-dependent smokers, assessed using the Fagerström Test for Nicotine Dependence (FTND > 7), in a single-arm pre-post design in which participants completed 20 supervised 30-minute sessions over four weeks. Feasibility was assessed through intervention fidelity, participant feedback, and instructor ratings, while preliminary effects were examined through changes in smoking urges and pulmonary function from baseline to four weeks.

Results

The final module retained nine yoga breathing practices that met the content validity threshold (CVR ≥ 0.31). Feasibility outcomes demonstrated a 100% acceptance rate and an 80% retention rate over the intervention period, with no adverse events reported. The instructor rated all practices as easy to learn and implement, while participants found them highly satisfactory, useful, and simple. At the end of four weeks, frequency and intensity of smoking urges showed significant reductions of 38.5% (p < 0.001) and 39.4% (p = 0.004), respectively, alongside a significant improvement in pulmonary function, with forced expiratory volume in 1 second (FEV₁) increasing by 17.98% (p < 0.001).

Conclusions

This study provides smokers with a structured, validated VYB module that is acceptable, safe, and feasible, with promising preliminary efficacy in reducing smoking-related urges, supporting cessation efforts, and improving pulmonary function. The findings highlight the potential clinical and public health relevance of this low-cost, nonpharmacological, breathing-only intervention for smoking cessation and pulmonary rehabilitation.

## Introduction

Cigarette smoking, the primary form of tobacco use, is the leading preventable cause of morbidity and premature death worldwide, causing over eight million deaths each year [1]. It is linked to a wide range of respiratory, cardiovascular, and oncological illnesses [2]. Smokers often experience chronic respiratory symptoms, higher rates of exacerbations like chronic obstructive pulmonary disease (COPD), signs of occult airway disease, airway wall thickening, and impaired pulmonary function [3]. In addition, smoking has been implicated in the pathogenesis of several neuropsychiatric conditions, including anxiety, depression, schizophrenia, dementia, attention-deficit/hyperactivity disorder, and suicidal behavior [4]. Long-term use has also been associated with cognitive decline, particularly impairments in executive function, working memory, and prospective memory [5]. Globally, smoking imposes an economic burden exceeding $1 trillion annually through healthcare costs and reduced productivity [6]. Developing nations experience a disproportionate burden of global tobacco consumption, with approximately 80% of the world’s 1.1 billion smokers residing in these regions. The prevalence of tobacco use continues to rise, a pattern reinforced by accelerating population growth and limited tobacco control measures [6,7].

Despite widespread awareness, many smokers relapse during quit attempts due to nicotine withdrawal symptoms such as persistent craving, irritability, attentional deficits, and negative affect [8]. Relapse often aggravates these symptoms, particularly low mood and stress, which further undermine quit motivation [9]. As elevated stress and emotional dysregulation are strong predictors of relapse, targeted interventions that support emotion regulation and craving management are crucial to improve cessation outcomes and sustain abstinence.

Yoga, a scientifically validated mind-body practice, integrates volitional yoga breathing (VYB; pranayama), which may be especially useful in smoking cessation. Established programs already recommend deep breathing techniques (e.g., diaphragmatic breathing) to reduce craving intensity during early abstinence [10]. Evidence further indicates that brief VYB practices, such as alternate-nostril and three-part breathing, can significantly reduce cravings [11]. Additionally, Bhastrika and deep breathing have been shown to alleviate stress, anxiety, and depression, which play a central role in smoking behavior and cessation success [12,13]. Moreover, these practices enhance self-regulatory capacity and promote prosocial emotional states and overall well-being [14].

Several studies have examined the efficacy of yoga breathing practices in reducing smoking-related symptoms and improving psychological health [10-12]. However, existing interventions lack consistency in their structure, content, and execution, and no standardized, standalone VYB module has been designed specifically for smokers. To address this gap, the present study developed and validated a structured yoga breathing module designed to address the physiological and psychological needs of smokers and assessed its feasibility, acceptability, and preliminary effectiveness in smoking cessation and pulmonary rehabilitation.

## Materials and methods

**Figure 1:**
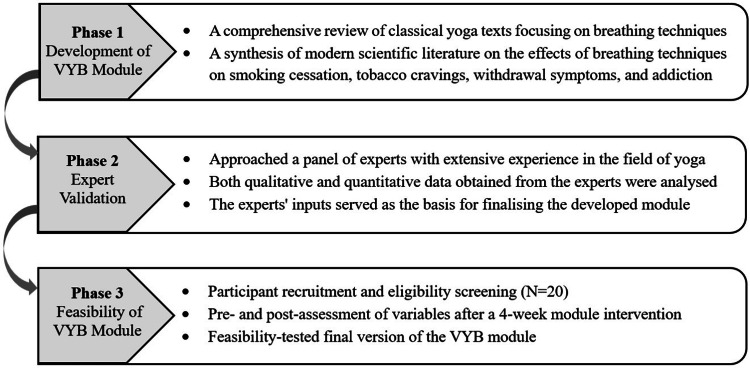
Three sequential phases of the proposed study VYB, volitional yoga breathing

The development, validation, and feasibility of the VYB module were conducted in three distinct phases, as illustrated in Figure 1.

Phase 1: Development of the VYB module

In this phase, a breathing module was developed specifically to help people quit smoking. The process began with a thorough review of four classical yoga texts: Gheranda Samhita [15], Yoga Vashishtha [16], Hatha Yoga Pradipika [17], and Shiva Samhita [18]. In addition, two contemporary yoga texts were examined: Asana Pranayama Mudra Bandha [19] and Science and Art of Yoga in Mental and Neurological Healthcare [20]. The review focused on identifying specific yoga breathing (pranayama) practices that may reduce nicotine cravings and withdrawal symptoms. These practices were also considered for their potential role in the pulmonary rehabilitation of smokers. Additionally, to complement the conventional sources, relevant scientific literature demonstrating the association between breathing practices and the targeted outcomes was reviewed using Google Scholar and the academic databases such as PubMed, Scopus, and Web of Science. Insights from yoga experts, psychologists, and psychiatrists were also incorporated during the development phase. Evidence from these sources emphasized the applicability of yoga breathing in healthcare and rehabilitation, facilitating the development of an evidence-based module rooted in tradition and supported by modern clinical research.

Finally, a comprehensive list of 11 yoga breathing practices was developed, based on evidence of their effectiveness in reducing nicotine cravings, easing withdrawal symptoms, and enhancing respiratory health and psychological well-being, while ensuring feasibility for the targeted population of smokers.

Phase 2: Expert validation

The developed module was emailed to a panel of 60 experts. The expert’s inclusion was based on the following eligibility criteria: (1) a postgraduate degree in yoga, complemented by a minimum of five years in teaching, research, or therapy within the field, or (2) a doctoral degree in yoga with a minimum of three years’ experience in yoga therapy and clinical research.

The validation form, administered via Google (Google LLC, Mountain View, CA, USA), provided a concise description of the study, its objectives, the practices included, and the rationale behind their selection. The evaluation of each practice consisted of two parts: a quantitative rating on a 3-point Likert scale (0 = Not necessary, 1 = Useful but not essential, and 2 = Essential) and a qualitative component for feedback on its relevance, duration, and sequence.

To assess content validity, we applied Lawshe’s content validity ratio (CVR) method [21]. For each practice, the CVR was calculated using the following formula:

$\mathrm{CVR} = \frac{N_e - N/2}{N/2},$

where Nₑ is the number of experts rating it “essential,” and N is the total number of experts.

Phase 3: Feasibility testing of the validated VYB module

We conducted a pilot pre-post study to evaluate the module’s feasibility with a sample of 20 smokers.

Participants and Settings

Twenty young adult males with high nicotine dependence were recruited from an addiction treatment center. Participants were included in the feasibility study if they (1) were aged between 18 and 45 years; (2) had no previous exposure to yoga; and (3) reported a nicotine dependence score greater than 7 on the Fagerström Test for Nicotine Dependence (FTND). Participants were excluded if they (1) had undergone surgery in the past one year; (2) had a low or moderate level of nicotine dependency (FTND score ≤ 7); (3) were using nicotine replacement therapy; (4) had chronic medical or neurological conditions such as diabetes, hypertension, or a history of stroke; (5) had a history of significant psychiatric conditions, for example, schizophrenia, bipolar disorder, or severe depression; (6) had severe visual or hearing impairments; (7) had co-occurring drug dependency (excluding alcohol); or (8) showed signs of complicated alcohol withdrawal.

Intervention

**Figure 2:**
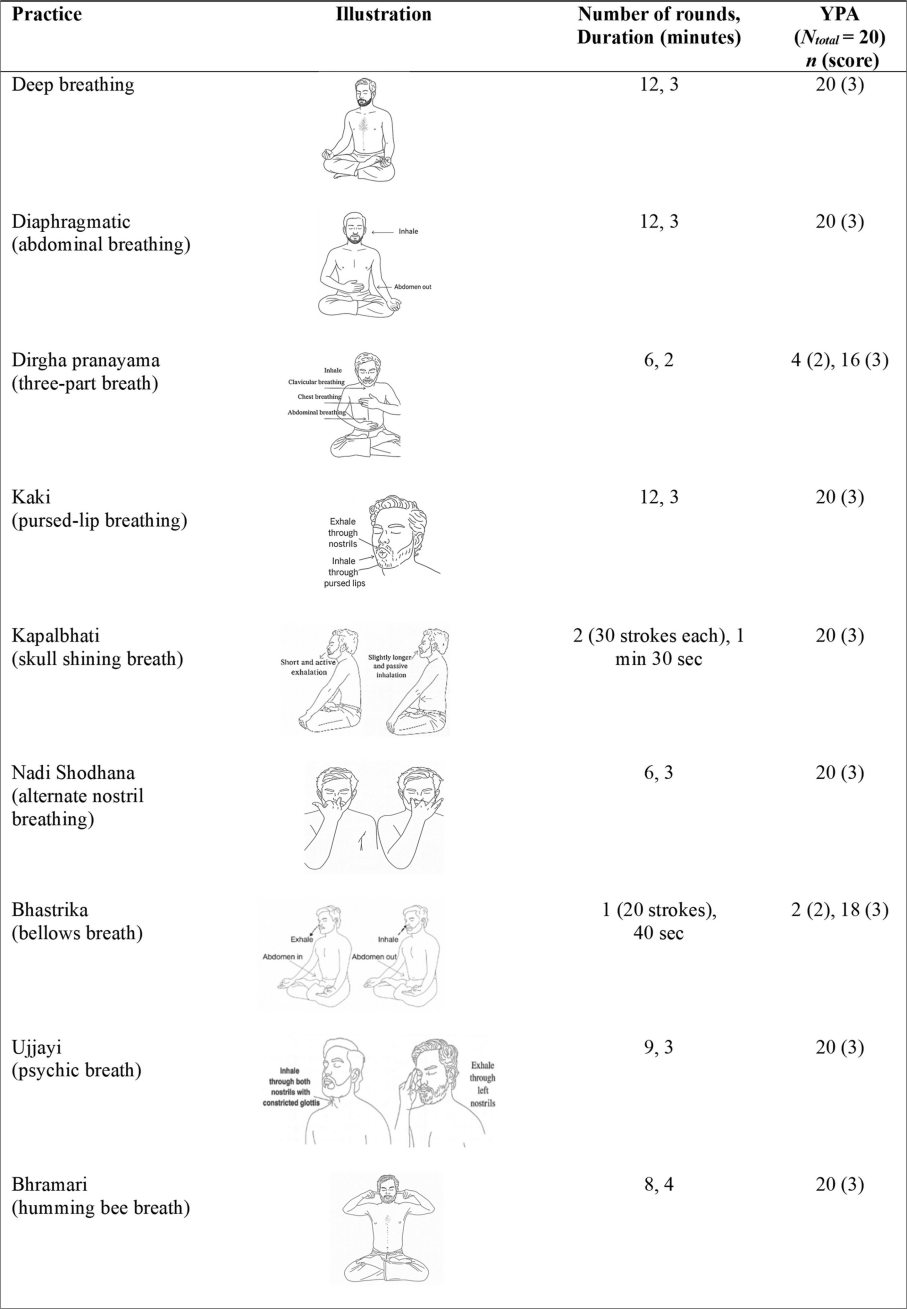
Final validated VYB module for smokers and YPA scores from the feasibility study 0 = can’t practice at all; 1 = needs assistance throughout the practice; 2 = needs assistance during some steps of the practice; 3 = able to perform the posture with ease VYB, volitional yoga breathing; YPA, Yoga Performance Assessment

The final validated VYB module was administered in a series of 20 evening sessions (6:00 to 6:30 p.m.) of 30 minutes each, conducted five days per week over four weeks under the supervision of a qualified yoga instructor. A one-minute rest was scheduled after each practice to restore normal breathing patterns. The Kapalbhati (high-frequency breathing) was administered in two cycles of 30 strokes each, with an additional 30-second interval between cycles. The first five sessions were slightly longer than 30 minutes, as the practices were administered more gradually due to participants’ lack of prior yoga experience. Details of the VYB intervention are provided in Figure 2.

Assessments

Feasibility was assessed using key measures of intervention fidelity, including acceptance rate, attrition (dropout) rate, retention rate, and any reported adverse events.

Face validity was evaluated in two ways. Participant feedback included ratings of practice usefulness on a four-point Likert scale and subjective evaluations of satisfaction, intention to continue, and perceived benefits on a three-point Likert scale (Appendices). Instructor-rated performance for each practice used the validated Yoga Performance Assessment (YPA) scale [22], a four-point scale from 0 (cannot practice at all) to 3 (can practice with ease without instructor assistance).

Outcome measures included (i) the two core items of the Mood and Physical Symptoms Scale (MPSS) to quantify smoking-related urges, such as frequency and intensity, each scored 0-5 (0 = not at all/no urges; 5 = all the time/extremely strong) [23] and (ii) the primary pulmonary function measure, forced expiratory volume in 1 second (FEV₁), measured using the RMS Helios 401 spirometer.

Statistical Analysis

Lawshe’s method was used to compute the CVR for each practice. Normality of the data was assessed, and paired t-tests were applied to evaluate changes in outcome measures using JASP software, version 0.19.3 (JASP Team).

Ethical Considerations

The study received approval from the Institutional Ethics Committee (CURAJ/H-IEC/25/03/0018, dated March 27, 2025) and was registered with the Clinical Trials Registry - India (CTRI/2025/04/084827, dated April 15, 2025). It was conducted in compliance with the Declaration of Helsinki, and all participants provided written informed consent before enrollment.

## Results

Phase 1: Development of the VYB module

Following an extensive review of classical and contemporary literature, research publications, and expert input, a VYB module consisting of 11 yogic breathing techniques was developed. These practices were chosen for their relevance and appropriateness to the intended objectives of the study (Table 1).

**Table 1:** VYB module for smokers with their CVR scores CVR, content validity ratio; VYB, volitional yoga breathing

Practice	Number of experts who responded			Number of expert responses with essential score (%)	CVR	Remarks
	1 (not necessary)	2 (useful but not essential)	3 (essential)			
Deep breathing	0	3	29	29 (90.6)	0.81	Retained
Dirgha pranayama (three-part breath)	1	5	26	26 (81.2)	0.62	Retained
Diaphragmatic breathing (abdominal breathing)	0	4	28	28 (87.5)	0.75	Retained
Kaki (pursed-lip breathing)	1	5	26	26 (81.2)	0.62	Retained
Chandrabheda (left nostril breathing)	2	11	19	19 (59.3)	0.18	Excluded
Suryabheda (vitality-stimulating breath)	2	15	15	15 (46.6)	-0.62	Excluded
Kapalbhati (skull shining breath)	1	2	29	29 (90.6)	0.81	Retained
Nadi Shodhana (alternate nostril breathing)	0	0	32	32 (100)	1	Retained
Bhastrika (bellows breath)	2	5	25	25 (78.1)	0.56	Retained
Ujjayi (psychic breath)	1	3	28	28 (87.5)	0.75	Retained
Bhramari (humming bee breath)	0	3	29	29 (90.6)	0.81	Retained

Phase 2: Expert validation

A total of 60 experts were approached through purposive and snowball sampling to validate the preliminary VYB module. Thirty-three responses were received, reflecting a 55% response rate, and one was excluded due to ineligibility. The inputs received from the final cohort of 32 experts were taken into consideration to validate and refine the module’s content, sequence, duration, and applicability for smoking cessation and pulmonary rehabilitation of smokers. Expert demographics are presented in Table 2.

**Table 2:** Demographic profile of the experts (n = 32)

Variable	n (%)
Gender composition	
Male	21 (65.6)
Female	11 (34.4)
Years of experience in the field, mean ± SD	12.40 ± 4.87
Education levels	
PhD	25 (78.1)
Postgraduation	7 (21.9)

Following Lawshe’s criteria, practices with CVR values ≥ 0.31, indicating significance at P < 0.05 (one-tailed, 32 experts), were retained in the final module. Two practices were excluded due to insufficient content validity (CVR < 0.31). Table 1 presents the experts’ ratings for each practice, their corresponding CVR values, and the resulting decision regarding retention in the final module. Based on these evaluations, nine practices were retained. Figure 2 illustrates the final selection of nine practices along with their respective rounds and durations.

Phase 3: Feasibility testing of the validated VYB module

Participant demographics are provided in Table 3.

**Table 3:** Demographic characteristics of smokers (N = 20) FTND, Fagerström Test for Nicotine Dependence

Variable	Value
Age, mean ± SD	26.5 ± 6.16
BMI ± SD	21.54 ± 2.86
Education level (above higher secondary), %	50 (n = 10)
Marital status (ever married), %	25 (n = 5)
Employed, %	40 (n = 8)
Smoking years, mean ± SD	10.70 ± 3.79
Cigarettes per day, mean ± SD	19.35 ± 6.81
Smoking quit attempts (past year), mean ± SD	2.8 ± 1.15
FTND score, mean ± SD	7.70 ± 1.12

Assessment of Intervention Fidelity

The study achieved a 100% acceptance rate, as all 20 invited individuals agreed to participate. Participants who attended a minimum of 14 sessions (70% of the total sessions) were considered for the post-assessment phase. The study achieved an 80% retention rate, with 16 out of the initial 20 participants completing the protocol. Notably, no adverse events were reported during the intervention period. These outcomes demonstrate that the intervention was delivered as intended and indicate strong feasibility, reflected in high participant engagement, retention, and safety.

Assessment of Face Validity

Participants’ responses on a 3-point Likert scale: Most participants (87.5%, 14/16) reported satisfaction with the intervention and indicated their intention to adhere to it in the future. All participants (100%, 16/16) reported a reduction in smoking urges and improvements in managing anxiety or restlessness associated with nicotine withdrawal. Additionally, 93.7% (15/16) found the intervention helpful in facilitating sleep, while 81.25% (13/16) experienced a reduction in the frequency of coughing or bringing up phlegm. Seventy-five percent (75%, 12/16) of participants reported decreased chest tightness or heaviness, and 87.5% (14/16) noted improvements in energy levels during daily activities as well as enhanced feelings of positivity and hope. Importantly, all participants agreed that the intervention was beneficial for smokers attempting to quit. The assessment items, adapted to evaluate these characteristics, are provided in the Appendices. In the checklist assessing perceived benefits of individual practices, all participants rated the practices as either “extremely useful” or “moderately useful.”

Instructor’s rating: The YPA ratings showed that most participants demonstrated the ability to learn and perform the VYB module by the end of the intervention (Figure 2).

Assessment of Clinical Outcome Measures

Statistical analysis: At the end of the 20 sessions, pre- and post-intervention data for frequency of urges, intensity of urges, and FEV₁ were compared using paired t-tests. The Shapiro-Wilk test indicated that all variables were normally distributed.

**Figure 3:**
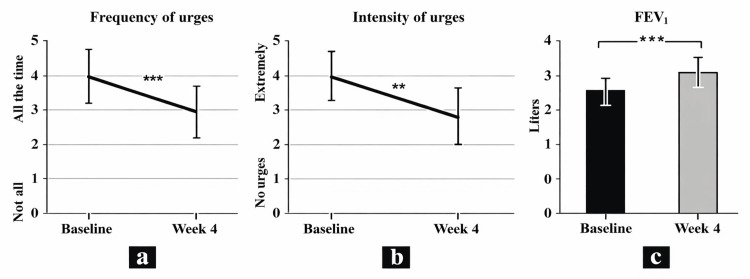
Changes in smoking urges and pulmonary function. (a) Changes in the frequency of urges to smoke on MPSS. (b) Changes in the intensity of urges to smoke on MPSS. (c) Changes in the FEV₁. ^**^ p < 0.01 ^***^ p < 0.001 Comparisons were made between pre- and post-test values. FEV₁, forced expiratory volume in 1 second; MPSS, Mood and Physical Symptoms Scale

Changes in smoking urges: At the four-week follow-up, participants reported a significant reduction in smoking urges compared with baseline. The frequency of urges to smoke decreased by 38.48% (p < 0.001), while the intensity of urges to smoke declined by 39.44% (p = 0.004) (Table 4, Figure 3).

**Table 4:** Assessment of outcome measures Paired t-tests were applied, with statistical significance set at p < 0.05; t = 5.92 for FEV₁, t = −5.58 for frequency of urges, and t = −3.37 for intensity of urges. FEV₁, forced expiratory volume in 1 second

Variable	Pre (mean ± SD)	Post (mean ± SD)	Change (%)	p-Value	Cohen’s d
Smoking urges					
Frequency of urges	3.95 ± 0.75	2.43 ± 1.03	38.48	<0.001	1.39
Intensity of urges	3.60 ± 1.09	2.18 ± 1.10	39.44	0.004	0.84
Pulmonary function					
FEV₁	2.28 ± 0.37	2.69 ± 0.47	17.98	<0.001	1.46

Changes in pulmonary function: Pulmonary function improved significantly after the 4-week intervention, with a 17.98% increase in FEV₁ (p < 0.001) (Table 4, Figure 3).

## Discussion

This study presents a validated VYB module consisting of nine practices aimed at supporting smoking cessation and pulmonary rehabilitation in smokers through reductions in nicotine urges and improvements in pulmonary function. The module was validated by 32 experts and tested for feasibility in 20 chronic smokers. It was found to be feasible, safe, and effective in reducing smoking urges and improving pulmonary function. It could be completed within 30 minutes, making it practical for routine implementation. Earlier interventions for smoking cessation include the Y-BLISS module, a brief 12-minute program that integrates yoga breathing techniques with relaxation and psychotherapeutic components [24]. This research addresses a clearly identified gap in the literature by, to our knowledge, being the first to evaluate the standalone efficacy of an intervention based exclusively on VYB. This study uniquely highlights VYB as a simple, cost-effective, and widely accessible intervention that is scalable, well-tolerated, and empowers individuals with self-applied techniques. Its portability and adaptability across settings position it as a public health strategy for both community and clinical use.

Smoking urges

The present study reported a statistically significant reduction in both frequency and intensity of the smoking urges. These findings extend earlier laboratory-based investigations that documented acute reductions in smoking urges following brief, single-session yoga breathing interventions lasting 10-20 minutes. In these studies, the effects were largely limited to the immediate post-intervention period or up to 24 hours and were observed predominantly among smokers with low levels of nicotine dependence (FTND < 5) [10,11]. The current study addressed these methodological limitations by employing a longer-duration, multi-session intervention administered over four weeks and specifically targeted smokers with high nicotine dependence (FTND > 7). Furthermore, implementing the intervention within a real-world addiction treatment setting enhances the clinical relevance and ecological validity of the findings. Taken together, the results support the efficacy of VYB across both controlled laboratory conditions and routine clinical settings.

Mechanistically, VYB may support smoking cessation by modulating craving-related processes. Slow, deep breathing enhances parasympathetic activity, reducing stress and negative affect, key triggers for relapse [25]. These practices have also been associated with emotional stability and reduced anxiety [26]. This autonomic shift, together with increased GABAergic activity [10], may account for the observed reductions in frequency and intensity of smoking urges. In addition, the rhythmic pattern of deep breathing may mimic the act of smoking and serve as a calming behavioral substitute, thereby helping to alleviate smoking urges [11,27].

Pulmonary function

The improvements in pulmonary function observed in the present study are consistent with findings from a prior single-group pre-post study that evaluated an eight-week yoga breathing-based intervention among young, asymptomatic smokers (six weekly sessions of alternate nostril breathing with Om chanting, 10 minutes each) [28]. That study reported a significant improvement in peak expiratory flow rate, along with improvements in respiratory endurance parameters; however, no significant changes were observed in FEV₁. Despite a shorter intervention duration of four weeks, the present study demonstrated a statistically meaningful improvement in FEV₁, a clinically important indicator of pulmonary function. This finding underscores the robustness of the VYB module, which was systematically structured, developed, and validated to address the specific needs of smokers. The focused, breathing-only design and standardized delivery of the VYB module may have enhanced intervention fidelity, thereby contributing to the observed pulmonary benefits.

VYB promotes parasympathetic activation and modulates sympathetic drive, improving autonomic balance and baseline airway tone. With regular practice, deeper lung expansion and prolonged inhalation stimulate pulmonary stretch receptors. This stimulation triggers a reflex reduction in tracheobronchial smooth muscle tone, which lowers airway resistance, increases airway caliber, and thereby enhances expiratory flow rates [28]. Concurrently, slow, controlled breathing facilitates reflex bronchodilatation, enhances surfactant release, and reduces airway inflammation; these effects act together to improve airway mechanics and overall pulmonary function [29].

This study is marked by many notable strengths. To our knowledge, this is the first study to validate a standalone yoga breathing module among smokers using a feasibility framework that incorporates both face validity and outcome measures. Significant improvements in outcomes, supported by participants’ self-assessments, demonstrate its effectiveness even over a brief intervention. The 30-minute module is adaptable and can be integrated with other therapies in rehabilitation centers and clinical settings, including pulmonary programs for asthma and COPD. Moreover, the module’s design allows for scalability in low-resource settings and delivery via telehealth or online platforms. It also has the potential for integration into national tobacco control programs, further enhancing its global relevance. The study highlights the promise of yoga breathing as a self-directed, cost-free, scalable, integrative approach for tobacco use disorder, strengthened by input from a multidisciplinary expert panel.

This study has several limitations: (1) the absence of a control group; (2) a small sample size and short intervention duration; (3) limited expert participation during content validation, with 32 out of 60 experts responding (~53%); and (4) the lack of long-term follow-up. Although attrition was 20% at 4 weeks, this reflects real-world challenges in addiction treatment centers, such as relapse, psychosocial stressors, limited family support, and the high cost of living and treatment, underscoring the importance of designing supportive systems to improve adherence. Despite these limitations, the findings provide preliminary evidence for the feasibility and potential of VYB in tobacco use disorder and pulmonary rehabilitation. Future randomized controlled trials with larger samples are needed to confirm effectiveness, alongside evaluations of integration into community-based cessation and rehabilitation programs and strategies to address implementation challenges in such contexts.

## Conclusions

This study provides smokers with a validated VYB module that is acceptable, feasible, and effective in reducing smoking urges and improving pulmonary function in chronic smokers. The findings offer preliminary evidence that VYB is a safe, low-cost, culturally acceptable, and scalable adjunctive strategy that can support smoking cessation and pulmonary rehabilitation. The simplicity of the intervention and its ease of integration into routine addiction treatment settings further enhance its feasibility and potential for broader clinical application. Future research should involve large-scale, multicenter randomized controlled trials with extended follow-up periods to confirm efficacy and inform the integration of this module into clinical practice and public health programs globally.

## Appendices

**Table 5:** Checklist for evaluation of subjective experience on the usefulness of VYB module

Practice	Not useful	Very little useful	Moderately useful	Extremely useful
Deep breathing	0	1	2	3
Diaphragmatic breathing (abdominal breathing)	0	1	2	3
Dirgha pranayama (three-part breath)	0	1	2	3
Kaki (pursed-lip breathing)	0	1	2	3
Kapalbhati (skull shining breath)	0	1	2	3
Nadi Shodhana (alternate nostril breathing)	0	1	2	3
Bhastrika (bellows breath)	0	1	2	3
Ujjayi (psychic breath)	0	1	2	3
Bhramari (humming bee breath)	0	1	2	3

**Table 6:** Self-reported participant feedback and perceived benefits of the VYB module

Questions	No	Somewhat	Yes
Are you satisfied with this yoga breathing program?	0	1	2
Do you plan to continue the practices from this program in the future?	0	1	2
Has the yoga breathing program helped in reducing your cigarette cravings?	0	1	2
Has the yoga breathing program helped in reducing your anxiety or restlessness?	0	1	2
Has the yoga breathing program helped you fall asleep more easily?	0	1	2
Has the yoga breathing program helped reduce how often you cough or bring up mucus?	0	1	2
Has the yoga breathing program helped reduce chest tightness or heaviness?	0	1	2
Has the yoga breathing program improved your energy or reduced fatigue during daily activities?	0	1	2
Has the yoga breathing program helped you feel more positive or hopeful?	0	1	2
Do you think the yoga breathing program can help smokers who are trying to quit smoking?	0	1	2

**Table 7:** Key domains of feasibility in the study

Domain	Meaning in context of this study	Outcomes (n=16)
Acceptability	Participants’ response to the VYB module	Satisfaction – 14 (87.5 %) Intention to continue use – 14 (87.5 %) Perceived appropriateness – 16 (100 %) Fit within organizational culture ‑ Yes Perceived positive or negative effects on organization – Not assessed
Demand	The extent to which participants are likely to adopt and continue using the VYB module	Actual use – Not assessed Expressed interest or intention to use – 14 (87.5 %) Perceived demand – Not assessed
Implementation	The extent to which the VYB module was administered successfully with participants in a defined, controlled setting	Degree of execution – All 9 breathing practices implemented without any modification Success or failure of execution - All 9 breathing practices implemented successfully Type and quantity of resources required for implementation – Qualified yoga instructor (s), yoga mats, and a hall or open space Factors influencing the ease and complexity of implementation – Instructor’s skill and experience eases implementation Implementation quality, speed, or efficiency – Participants being healthy young adult smokers facilitates speedy and efficient implementation of intervention
Practicality	The extent to which the VYB module could be conducted within the available time and resources	Positive effects on target population – 16 (100 %) Negative effects on target population – Nil Ability of participants to perform intervention tasks – Easy to execute, though initial sessions took more than 30 min Cost analysis – Not applicable as the intervention being cost-free
Adaptation	Adjusting the VYB module delivery to suit individual needs, capabilities, and religious contexts	Modifications/adaptations – No modifications were made to the original steps of the practices; however, the initial sessions were conducted at a slower pace
Integration	The extent to which VYB module can be integrated within established addiction treatment facilities or controlled settings.	The intervention, conducted in a hall within an addiction treatment centre; therefore, this module can be integrated into existing de-addiction centres or other controlled settings. Perceived fit with infrastructure – Yes Perceived sustainability – Not assessed Costs to organization and policy bodies ‑ Not assessed
Expansion	The scope for designing a new program through the expansion of the existing yoga breathing program	The possibility of expansion was not assessed, as this was the first instance of executing the VYB module Fit with organizational goals and culture – Yes Positive or negative effects on organization ‑ Not assessed Disruption due to expansion components – Not assessed
Limited-efficacy	Employing a convenience sample, the evaluation explored the potential effectiveness of the VYB module for smokers	Intended of program or process on key intermediate variables – Yes, significant reduction in smoking cravings and improvement in pulmonary function measure Effect size estimation – Yes Maintenance of changes from initial change – No follow-up
